# Hepatitis B prevalence in Brazilian waste pickers: a systematic review with meta-analysis

**DOI:** 10.11606/s1518-8787.2021055003115

**Published:** 2021-11-16

**Authors:** Gabriel Souza-Silva, Marcos Paulo Gomes Mol

**Affiliations:** I Fundação Ezequiel Dias Diretoria de Pesquisa e Desenvolvimento Belo Horizonte MG Brasil Fundação Ezequiel Dias. Diretoria de Pesquisa e Desenvolvimento. Belo Horizonte, MG, Brasil

**Keywords:** Solid Waste Segregators, Hepatitis B, epidemiology, Systematic Review

## Abstract

**OBJECTIVE:**

To describe the hepatitis B prevalence in Brazilian waste pickers.

**METHODS:**

We performed a literature search in the SciELO, Biblioteca Virtual em Saúde (BVS), PubMed and Web of Science databases using the descriptors: “hepatitis B” AND (“informal recycling” OR “waste picker” OR “recyclable waste collectors” OR “solid waste segregator”) AND (“recyclable waste” OR “solid waste”) AND Brazil. Epidemiological studies on HBV in Brazilian waste pickers published prior to February 2020 were included and evaluated for quality and bias using a funnel plot.

**RESULTS:**

This meta-analysis consisted of five articles. Prevalence of HBV surface antigen seropositivity was 14% (95%CI: 6%–22%) in Brazilian waste pickers.

**CONCLUSION:**

HBV prevalence in Brazilian waste pickers remains high. There should be more campaigns showing the importance of vaccination and personal protective equipment use.

## INTRODUCTION

According to the World Health Organization (WHO), hepatitis B virus (HBV) infection can cause acute or chronic liver diseases, causing liver cirrhosis, and can be associated through contact with contaminated blood and other body fluids. Hepatitis B diagnostic trials are based on HBV surface antigen (HBsAg) detection^1^. HBV infection has a 3.9% prevalence worldwide and was responsible for 887,000 deaths in 2015^1,2^.

Between 1999 and 2018, Brazil confirmed 233,027 new cases of hepatitis B distributed in the Southeast (34.9%), South (31.6%), North (14.4%), Northeast (9.9%) and Midwest (9.1%)^3^. Although HBV vaccine is available for free and provided to all newborns and high-risk individuals since 1999, hepatitis B rates in Brazil have shown only small variations, with a slight downward trend in recent years, with 8.7 cases per 100,000 inhabitants in 2011 and 6.7 cases per 100,000 inhabitants in 2018^3^.

HBV infection can lead to serious complications, impairing the organ’s functional capacity and, in more severe cases, interrupting its vital functions. Chronicity of the disease increases the risk of developing liver cancer, kidney disease, or inflammation of the blood vessels^4^.

In Brazil, an estimated number of one million waste pickers represent 31% of the homeless population, whose main activity is collecting recyclable materials in an individual, informal, and usually autonomous way^5^. Inmates and injecting drug users may also be considered groups vulnerable to hepatitis B virus infection^6^.

Despite their high vulnerability to HBV infection, data on vulnerable populations such as waste pickers regarding HBV prevalence is still scarce. According to Souza-Silva and Mol (2020)^7^, few studies in Latin America address this topic, with Brazil being the only country with data on HBV prevalence in waste pickers. 

These workers mainly collect materials from garbage cans, the streets, and domestic, industrial, hospital and commercial sources for sale, recycling or reuse for their own consumption^8^. During collection, they are constantly exposed to physical (cuts and mutilations), chemical (heavy metal contamination or toxic products), and biological (human immunodeficiency virus, hepatitis and other infectious diseases) risks caused by contact with sharp items, besides dealing with social exclusion due to their socioeconomic status^9,10^.

Due to their activities, waste pickers are vulnerable to occupational risks, poverty, cultural issues and improper working environment, and thus vulnerable to hepatitis B infections. Factors such as inadequate waste handling and lack of personal protective equipment (PPE) use also contribute to this increased HBV prevalence^8,11^. Based on this context, this systematic review with meta-analysis sought to describe the prevalence of hepatitis B in waste pickers of recyclable material in Brazil, providing a clearer and more comprehensive view of published data on HBV prevalence in these informal workers.

## METHODS

All steps of this systematic review with meta-analysis complied with the Preferred Reporting Items for Systematic Reviews and Meta-Analyses (PRISMA) checklist^12^ and Meta-analyses of Observational Studies in Epidemiology (MOOSE) guidelines^13^.

We performed a literature search on the SciELO, Biblioteca Virtual em Saúde (BVS), PubMed and Web of Science databases using the following descriptors: “hepatitis B” AND (“informal recycling” OR “waste picker” OR “recyclable waste collectors” OR “solid waste segregator”) AND (“recyclable waste” OR “solid waste”) AND Brazil. Regarding year of publication and language, we included any study published prior to February 2020 written in English or Portuguese. Articles were first selected by reading of their titles and abstracts; those that met the inclusion criteria were read in full.

To maximize the literature search, we used the Google Scholar aggregator to scan gray literature. After tabulation of all articles found, both authors (GSS and MPGM) performed a blind selected by reading their titles and abstracts; those included for full-text reading were obtained by consensus. Finally, we extracted data on the prevalence of hepatitis B virus infection in Brazilian waste pickers.

Inclusion criteria consisted of: hepatitis B; epidemiological study (prevalence, seroprevalence or other epidemiological study); hepatitis B prevalence results; conducted in Brazil; results on (informal) waste pickers. The exclusion criteria comprised: not conducted in Brazil; language other than English or Portuguese; not conducted with waste pickers; duplicate; topic other than hepatitis B; not an epidemiology study.

Both authors performed a blind quality evaluation for each selected study, attributing a score ranging from 0 to 8 based on the Loney criteria^14^, which includes: 1. random sample or whole population; 2. unbiased sampling method (inclusion criteria described); 3. adequate sample size (sample calculation method described); 4. measures used standard methods (as recommended for epidemiological studies); 5. outcomes measured by unbiased evaluators (methods to control bias described); 6. adequate response rate, refusals described (response rate adequate according to the sample); 7. confidence intervals, subgroup analysis (adequate statistical methods); 8. study subject described. Each item equals one point.

General information (authors, year, place) and primary data (study design, sample size, number of waste pickers with and without HBV, number of positive HBsAg and anti-HBc cases) were extracted from each article included in the meta-analysis. We calculated both the standard error and the standard deviation for all variables.

All analyses were conducted using the metafor package for R software, version 3.5.3. HBV serology results were presented individually (Box), with most studies using HBsAg to identify incubated, acute or chronic diseases. Publication bias was evaluated using a funnel plot and confirmed by Egger’s linear regression test. The main prevalence results were estimated using a forest plot. Heterogeneity between selected articles was calculated using Chi-Square test based on Q statistic and random effect model (I^2^ = 96,34%; Cochran’s Q = 109.22, p < 0.0001). Prevalence of HBsAg infection was calculated as 95% conﬁdence intervals (CI) and used as summary measures in all meta-analyses.

**Box:** General data of studies included in this meta-analysis and complete quality criteria adopted (papers presented according to the more recent year of publication).

Year of publication	Reference	Year of study	Place	Study design	Sample size (n)	HBV prevalence (%)^a^	Quality (total score)	Limitations^b^
2008	Rozman MA et al.^20^	2005	Santos (SP)	Cross-sectional	250	33.4	8	Not applicable
2014	Marinho TA et al.^21^	2010–2011	Goiânia (GO)	Cross-sectional	431	12.8	7	Outcomes measured by biased assessors
2019	Cruvinel VRN et al.^16^	2017	Brasília (DF)	Cross-sectional	770	4.3	6	Outcomes measured by biased assessors and inadequate response rate
2019	Klein G et al.^22^	2015	Jardim Gramacho (RJ)	Cross-sectional	73	12.3	5	Outcomes measured by biased assessors, inadequate sample size and inadequate response rate
2020	Weis-Torres SMS et al.^23^	2014–2016	Campo Grande (MS)	Cross-sectional	278	10.1	7	Outcomes measured by biased assessor

a Hepatitis B showed general prevalence when HBsAg and Anti-HBc were used.

b According to guidelines for critically appraising studies on prevalence or incidence of a health problem (Loney et al.^14^).

## RESULTS

**Figure 1:**
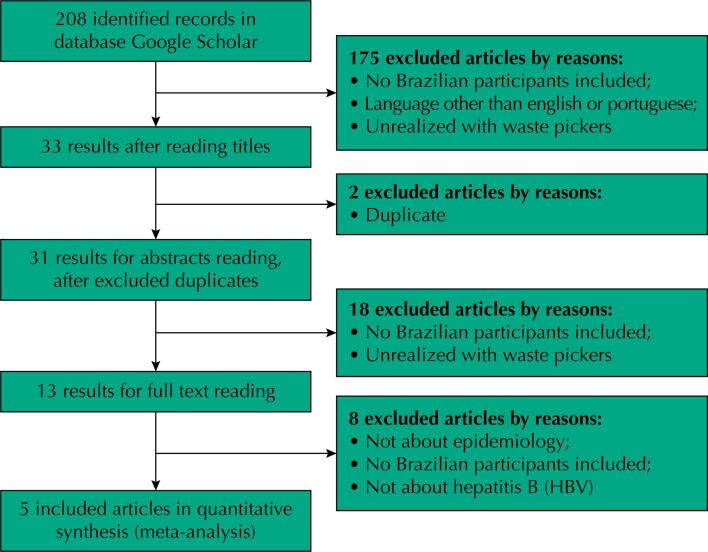
Flowchart of the search and inclusion papers, per stages.

The literature search returned 208 articles, of which 33 remained after reading the titles and deleting duplicates. After screening the abstracts, we excluded other 18 papers (study not conducted in Brazil or not carried out with waste pickers). From the 13 studies selected to be read in full, only 5^8–11,15^ remained in the meta-analysis (Figure 1, Box).

All studies had a cross-sectional design and used the enzyme-linked immunosorbent assay (ELISA) to detected HBsAg. Of the 1,802 participants included in the meta-analysis, 208 (11.54%) tested positive for HBV infection. The selected studies described HBV epidemiological data with prevalence ranging from 4.3 to 33.4% for all HBV markers (HBsAg and Anti-HBc). Regarding quality evaluation, the articles included showed an average score of 6.6 points. For the score per paper, see the Box.

**Figure 2:**
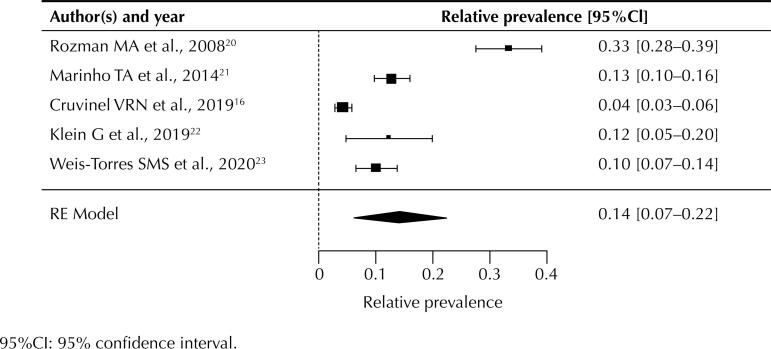
Prevalence of HBV infection among HBsAg-positive carriers.

**Figure 3:**
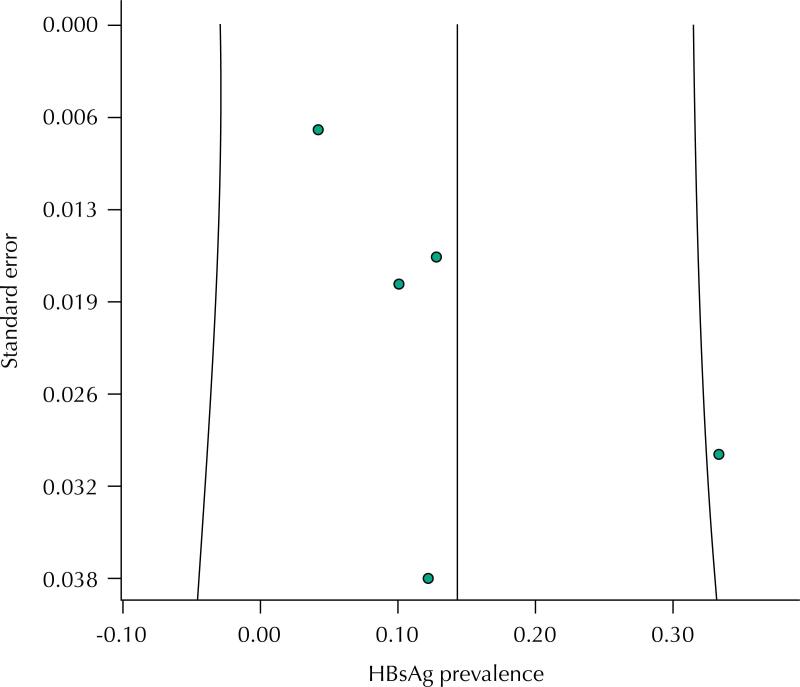
Funnel-plot of papers selected to meta-analysis, according to HBsAg results.

Figure 2 shows the distribution of HBsAg positive serology among waste pickers, with a HBsAg seropositivity rate of 0.14 (95%CI: 0.06–0.22). The funnel plot (Figure 3) found no asymmetry, indicating absence of publication bias, which we confirmed using linear regression analysis (p = 0.088).

As heterogeneity was found between the papers (p < 0.0001), we used a random effect model. Used to evaluate the significance of this heterogeneity, the chi-square test statistically proved the methodological similarity of the papers selected, thus validating the combination of their results.

## DISCUSSION

The scarcity of studies on hepatitis B prevalence in Brazilian waste pickers reinforces the importance of the present meta-analysis, as a means to provide a better understanding about the current prevalence of HBV infection among this group. Alongside other neglected populations such as prisoners, homeless individuals, drug addicts and sex workers, waste pickers are at risk of HVB infection^16^ due to their social and economic vulnerability, presenting high prevalence values of the disease when compared to the general population^11^.

Although the Brazilian National Immunization Program has included free hepatitis B vaccine since 1999, the number of hepatitis B cases have virtually stayed the same^3^, probably due to social negligence regarding vaccination and transmission among unvaccinated youth and adults. Studies show that lower-income families present greater HBV vulnerability, having both low schooling levels and low levels of vaccination against HBV^9,11,15^.

Hepatitis B vaccination is obligatory only for healthcare workers, including waste workers that serve healthcare facilities. Urban solid waste collectors and waste pickers, although also exposed to potential infected wastes, remained excluded^17^.

Exposure to sharp instruments, such as glass shards, and lack of protective equipment (boots, gloves and masks) during waste collection, and poor access to information increases the risk of hepatitis B infection among waste pickers when compared to the general population^18,19^. Another contributing factor to this increased vulnerability is the dumping site, which is conducive to diseases and infections caused by vectors such as insects and rats, or though contact with contaminated waste or water^8,9^.

In Brazil, the prevalence of Anti-HBc in the general population between ages 20-69 years is 11.6%, varying according to region: North (14.7%), Northeast (11.7%), Midwest (12.7%), Southeast (7.9%), South (11.3%). In adolescents (between 10-19 years old), these rates do not exceed 2.1%^20^. The values are even lower regarding the prevalence of virus carriers (HBsAg positive), not exceeding 1% for the ages 20-69 years and reaching only 0.2% for individuals aged 10 to 19 years old, in any region of the country^20^. Economic and social factors may influence the increase or decrease of this prevalence^21^.

The present meta-analysis estimated a combined HBV prevalence in waste pickers of 14% (95% CI: 6%–22%). Brazil is traditionally recognized as a country of intermediate endemicity for hepatitis B, with great heterogeneity between its regions. Considering the vulnerability of waste pickers to HBV infection, we expected the prevalence of this virus to be higher. Such finding may be explained by the selection biases observed during the literature review, since the studies on HBV prevalence in waste pickers present data for certain regions in Brazil.

Studies conducted in Nigeria^22^, Pakistan^23^ and Central Greece^24^ showed HBV prevalence rates in waste pickers of 17.4%, 18.8% and 23.0%, respectively. Regarding the potential causes of hepatitis B infection in waste pickers, the papers suggested: handling waste with bare hands and feet; inadequate and precarious waste disposal system; poor vaccination status; handling of piercing-cutting instruments (biomedical waste); no use of personal protective equipment; health insurance; unavailability of health education material.

Despite performing a marginal and informal work, waste pickers contribute positively to the recycling process and waste reduction, reducing collection and transportation costs and the import of raw materials. Being an informal job, reports of health problems are quite common; for example, even with increased awareness of hepatitis B in the general Brazilian population, this meta-analysis found higher HBV prevalence in waste pickers. These findings indicate the need for increased vaccination against HBV and the adoption of strategies to prevent HBV infection in this population^25^.

### Limitations

Even including a small number of papers, our study highlighted the poor scientific discussion regarding hepatitis B prevalence in waste pickers. To our knowledge, no meta-analysis on the HBV prevalence in Brazilian waste pickers has been published. All included papers presented serologically confirmed HBV infection data, but the small number of studies available on this topic imposed statistical limitations. We adopted all methodological steps to validate our results, including heterogeneity and bias statistical tests.

## CONCLUSION

HBV prevalence in Brazilian waste pickers remains high and above that of the general Brazilian population, having similar rates to countries such as Nigeria and Pakistan. More studies are needed on the health of Brazilian waste pickers, especially ones discussing the context of hepatitis B. Policies aimed at increasing HBV vaccination rates and implementing awareness actions on the importance of using personal protection equipment during waste collection are also necessary.

We also highlight the importance of increasing hepatitis B vaccination in Brazil, particularly by reviewing the current healthcare policies to include all waste workers. Health protection is the key to promoting public health.
